# Novel circGFRα1 Promotes Self-Renewal of Female Germline Stem Cells Mediated by m^6^A Writer METTL14

**DOI:** 10.3389/fcell.2021.640402

**Published:** 2021-04-12

**Authors:** Xiaoyong Li, Geng Tian, Ji Wu

**Affiliations:** ^1^Key Laboratory for the Genetics of Developmental and Neuropsychiatric Disorders (Ministry of Education), Renji Hospital, School of Medicine, Bio-X Institutes, Shanghai Jiao Tong University, Shanghai, China; ^2^Key Laboratory of Fertility Preservation and Maintenance of Ministry of Education, Ningxia Medical University, Yinchuan, China

**Keywords:** circGFRα1, METTL14, ceRNA, female germline stem cells, self-renewal

## Abstract

Circular RNAs (circRNAs) play important roles in the self-renewal of stem cells. However, their significance and regulatory mechanisms in female germline stem cells (FGSCs) are largely unknown. Here, we identified an *N*^6^-methyladenosine (m^6^A)-modified circRNA, circGFRα1, which is highly abundant in mouse ovary and stage-specifically expressed in mouse FGSC development. Knockdown of circGFRα1 in FGSCs significantly reduced their self-renewal. In contrast, overexpression of circGFRα1 enhanced FGSC self-renewal. Mechanistically, circGFRα1 promotes FGSC self-renewal by acting as a competing endogenous RNA (ceRNA) that sponges miR-449, leading to enhanced GFRα1 expression and activation of the glial cell derived neurotrophic factor (GDNF) signaling pathway. Furthermore, circGFRα1 acts as a ceRNA based on METTL14-mediated cytoplasmic export through the GGACU motif. Our study should help to understand the mechanisms regulating germ cell development, add new evidence on the mechanism of action of circRNA, and deepen our understanding of the development of FGSCs.

## Introduction

The infertility rate globally has increased year by year, with an average incidence of 12.5%. Infertility has become the third most common disease threatening human health, after cardiovascular disease and cancer. A shortage and poor quality of oocytes are key factors leading to female infertility. There is thus an urgent need to understand the mechanisms of female reproduction in order to improve the quantity and quality of oocytes. As germline stem cells, female germline stem cells (FGSCs) can increase oocyte number and improve ovarian function, which is of great significance in mammals to improve the quality of oocytes and the pregnancy rate (Zou et al., 2009). These cells are thus becoming a focus of medical care.

With the deepening of the research, great progress has been made in the self-renewal of FGSCs. For example, a series of genes and signal pathways affecting FGSC self-renewal have been identified, such as STPBC, AKT1, AKT3, glial cell derived neurotrophic factor (GDNF) signaling pathway, phosphoinositide-3 kinase–AKT (PI3K–Akt) signaling pathway, Hippo signaling pathway, and Notch signaling pathway (Xie et al., 2014; Li et al., 2015, 2017a, 2019; Pan et al., 2015; Zhang et al., 2016, 2018; Liu et al., 2017; Ma et al., 2018; Zhu et al., 2018; Wu et al., 2019). GDNF signaling acts by modifying the activity of PI3K–AKT mitogen-activated protein kinase/ERK kinase, and the Src family of downstream substrates, ultimately affecting the expression of genes such as Bcl6b, Lhx1, Etv5, and Egr3.

The main characteristics of circular RNAs (circRNAs) are as follows. Most of circRNAs exist in the cytoplasm, but a small part of circRNAs formed by intron cyclization exist in the nucleus; circRNAs widely exist in human cells, sometimes more common than linear RNA; circRNAs are closed loop structure, not easy to be degraded by RNaseR; circRNAs are highly conservative; most of circRNAs are formed by exon cyclization, and a small part are formed by intron cyclization; Some of circRNAs play the role of miRNA sponge in cells, and a few of them can be translated into proteins (Burd et al., 2010; Memczak et al., 2013; Sun et al., 2013; Zhang et al., 2013). Compared with the miRNA regulatory network, the competing endogenous RNAs (ceRNAs) regulatory network is more sophisticated and complex, involving more RNA molecules, including mRNA, pseudogenes encoding genes, circRNA, lncRNA, and microRNA (Hansen et al., 2013; Zhang et al., 2013; Guo et al., 2014; Ahmed et al., 2016). However, to date, no circRNAs critical in the development of FGSCs, or their functions and/or underlying mechanisms, have been discovered.

*N*^6^-methyladenosine (m^6^A) is the most important modification in mRNA epigenetics. Similarly, in the circRNA epigenetic transcriptome, m^6^A is also a highly abundant, widely distributed, and functionally important post-transcriptional modification (Shafik et al., 2016; Jacob et al., 2017). Yang et al. (2017) showed that some circRNAs could also recruit translation initiation complexes to start translating proteins by binding with the YTHDF3 recognition protein. In addition, Wu et al. (2019) found that circ_KIAA1429 accelerates the progression of hepatocellular carcinoma through m6A-YTHDF3-Zeb1.

In this study, on the basis of genome-wide circRNA analysis (Li et al., 2019), we identified a novel circRNA, circGFRα1, the biological function in FGSC development of which has not yet been clarified. We found that circGFRα1 was highly abundant in mouse ovary and stage-specifically expressed in mouse FGSC development. Importantly, circGFRα1 promoted the self-renewal of FGSCs. Mechanistically, we found that METTL14-mediated m^6^A modification altered circGFRα1 export to the cytoplasm, while circGFRα1 acted as a ceRNA to regulate GFRα1 expression by sponging miR-449 to play regulatory roles in FGSC development. Our findings reveal a novel mechanism regulating FGSC self-renewal and provide a theoretical basis for the study of germ cell development and human reproduction.

## Materials and Methods

### Culture of Female Germline Stem Cells

FGSCs were cultured according to the previously method (Zou et al., 2009; Zhang et al., 2016; Li et al., 2017b). FGSCs passages were performed at a ratio of 1:4 and intervals of 4–7 days.

### Plasmid DNA

We used pLCDH-ciR to construct the circGFRα1 overexpression vector to perform transcript circularization. An *Eco*RI restriction enzyme site was discovered within an endogenous flanking sequence located in the front circular frame. On the other hand, a *Bam*HI site was detected within a partially inverted upstream sequence located in the back circular frame. We cloned the fragment amplified into the vector between the front and the back circular frames. We also constructed a control vector that contained only a non-sense insert between the front and back circular frames in the absence of circGFRα1-encoding cDNA. Lentiviral vectors (pGMLV-SC5) loaded with anti-circGFRα1 shRNA and the negative control shRNA (nc-shRNA) with EGFP were provided by Shanghai Genomeditech Company Ltd. In the meantime, we used an irrelevant, scrambled shRNA that was not matched with the mouse genome sequence as the control.

For constructing the METTL14-knockdown lentiviral vectors, we applied molecular biological approaches to insert an interfering fragment in the U6 promoter downstream into the lentiviral vector (pLKD-CMV-G and PR-U6-shRNA). We selected four or more independent siRNAs to examine the target knockdown efficiency. Finally, we selected the optimal siRNA target (targetSeq: GCTAAAGGATGAGTTAAT). To construct METTL14 overexpression vectors, we inserted the candidate gene cDNAs into the *Bam*HI and *Eco*RI restriction sites in an overexpression plasmid (pHBLV-CMVIE-ZsGreen-T2A-puro).

### Viral Preparation and Transduction

We used 293T cells to prepare lentiviruses according to a method described previously (Shi et al., 2004). For lentivirus infection, the FGSCs were incubated with 1:1 mixture of culture medium and lentivirus (titer: 2.5 × 10^8^). After 12–16 h of infection, the culture medium containing lentivirus particles was sucked out, and the culture medium was added to the culture plate for further culture. Subsequently, the FGSCs were screened by 100 ng/ml puromycin.

### RNA Fluorescence *in situ* Hybridization Assay

The RNA-FISH procedure for circGFRα1 was performed with the RNA-FISH kit of GenePharma Inc. (Shanghai, China), according to the manufacturer’s instructions. Briefly, FGSCs were cultured in 48-wells overnight at 37°C and 5% CO2. Next day, cells were rinsed in chilled phosphate-buffered saline (PBS) two times. Cells were fixed with 4% paraformaldehyde in the room temperature for 30 min. Then 0.1% buffer A was added and incubated in the room temperature for 30 min. After that, the cells were washed twice with PBS for 5 min. The RNA-FISH probes targeting circGFRα1 were synthesized by GenePharma Inc. (Shanghai, China). Those probes were mixed well with buffer E to a final concentration 2 μM, then to hybridize with cell’s genes and incubate overnight at 37°C. The cells were washed twice with buffer C for 5 min. This was followed by rinsing and staining with DAPI (1:1,000 dilution; Sigma)-containing PBS at room temperature for 5 min. The images were acquired with a fluorescence microscope (Leica, United States).

### CCK8 Assay

We cultured FGSCs in each well (containing 200 μL culture medium) of 96-well plates at a density of 5,000 cells/well. At a confluence of 70%–80%, we added 20 μL of the CCK8 reagent into each well of the plate and incubated it for another 2 h at 37°C and 5% CO_2_. Then, we used a microplate reader to measure the absorbance (OD) value at 450 nm.

### EdU Assay

We cultured FGSCs to 80% confluence and added 50 μM of the EdU reagent into each well, after which the plate was incubated for another 2 h. Thereafter, cells were fixed with 4% PFA for 30 min under ambient temperature and neutralized for 5 min by using 2 mg/mL glycine. Then, the cells were punched with 0.5% Triton X-100, stained with the 1 × Apollo staining solution, incubated for 30 min, and finally washed thrice by using PBS supplemented with 0.5% Triton X-100. Finally, cell nuclei were dyed using 1 × Hoechst 33342. A Leica fluorescence microscope was used to capture images.

### m^6^A Dot Blot

Trizol reagent was used to extract total RNA from FGSCs. After denaturing at 95°C, the RNAs were immediately chilled on ice. Then, they were dropped onto a Hybond-N + membrane. The membrane was cross-linked with a UV cross-linker, followed blocking with 5% skim milk, and overnight incubation with the m6A-specific antibody (1: 1,000) at 4°C. Thereafter, the membrane was rinsed by TBST for 10 min, followed by another 1 h of incubation with a secondary antibody at an ambient temperature. Finally, Tanon 4600SF was used to scan the dots.

### qRT-PCR

Trizol reagent was used to extract total cellular RNA from FGSCs that was quantified using Nanodrop Lite. cDNA was prepared by reverse transcribing the RNA (1,000 ng) by using a reverse transcription kit in the 20-μL system. In this qRT-PCR assay, Taq DNA polymerase was used with SYBR Premix Ex Taq in the Applied Biosystems^®^ 7500 Real-Time PCR system. The 2^–ΔΔCt^ approach was used for data analysis.

### MeRIP-qPCR

Total RNA was extracted from FGSCs by using the Trizol reagent and quantified using Nanodrop Lite. We bound 1.25 μg of the anti-m6A antibody onto protein A/G magnetic beads dissolved in the IP buffer (consisting of 140 mM NaCl, 20 mM Tris pH 7.5, 2 mM EDTA, and 1% NP-40) 1 h in advance. Thereafter, we incubated the resultant antibody-bound protein A/G beads with the RNA sample at 4°C for 2 h. Then, the obtained samples were rinsed twice by using low-salt wash buffer (composed of 5 mM EDTA and 10 mM Tris; pH 7.5), followed by washing with a high-salt wash buffer (composed of 1 M NaCl, 20 mM Tris pH 7.5, 0.5% sodium deoxycholate, 1% NP-40, 1 mM EDTA and 0.1% SDS) twice and then with the RIPA buffer (150 mM NaCl, 20 mM Tris pH 7.5, 0.5% sodium deoxycholate, 1% NP-40, 1 mM EDTA, and 0.1% SDS) twice. For all the samples, their wash solutions were harvested and considered the EDTA, an fraction. In addition, the beads were incubated with 50 μL *N*^6^-methyladenosine 5-monophosphate sodium salt (20 mM) at 4°C for 1 h to elute the RNA. After precipitation with ethanol, cDNA was prepared by reverse transcribing the RNA in the input, unbound and m6A-bound fractions by using Superscript III random hexamers. Subsequently, qRT-PCR was performed to determine the m6A-containing transcript levels compared with the Rplp0 level. For Rplp0, the primer sequences were GATGGGCAACTGTACCTGACTG and CTGGGCTCCTCTTGGAATG.

### Dual-Luciferase Reporter Assay

We inserted the mutant (circGFRα1-MUT) and wild-type (circGFRα1-WT) circGFRα1 miRNA-binding site sequences into *Sac*I and *Kpn*I sites in the pGL3 promoter vector. Thereafter, we cultured the cells in 24-well plates and used Lipofectamine 3000 to transfect 5 ng of the pRL-SV40 *Renilla* luciferase vector and 80 ng plasmid, together with 50 nM of the negative control or miR-449 mimics into the cells. After 48 h, we harvested and examined the cells by performing the dual-luciferase assay according to specific protocols. Each independent experiment was performed thrice.

### Statistical Analysis

The significance of difference in graphs was assessed using Student’s *t*-test unless specified otherwise. The normally distributed two-sample equal variance was used in this study. Our researchers were aware of sample grouping in each experiment. *P* value of <0.05 indicated statistical significance. Graphs as well as error bars are represented as means ± SEM unless specified otherwise. The R statistical environment and GraphPad Prism 4.0 were used for performing statistical analysis.

## Results

### Identification and Characterization of circGFRα1

**FIGURE 1:**
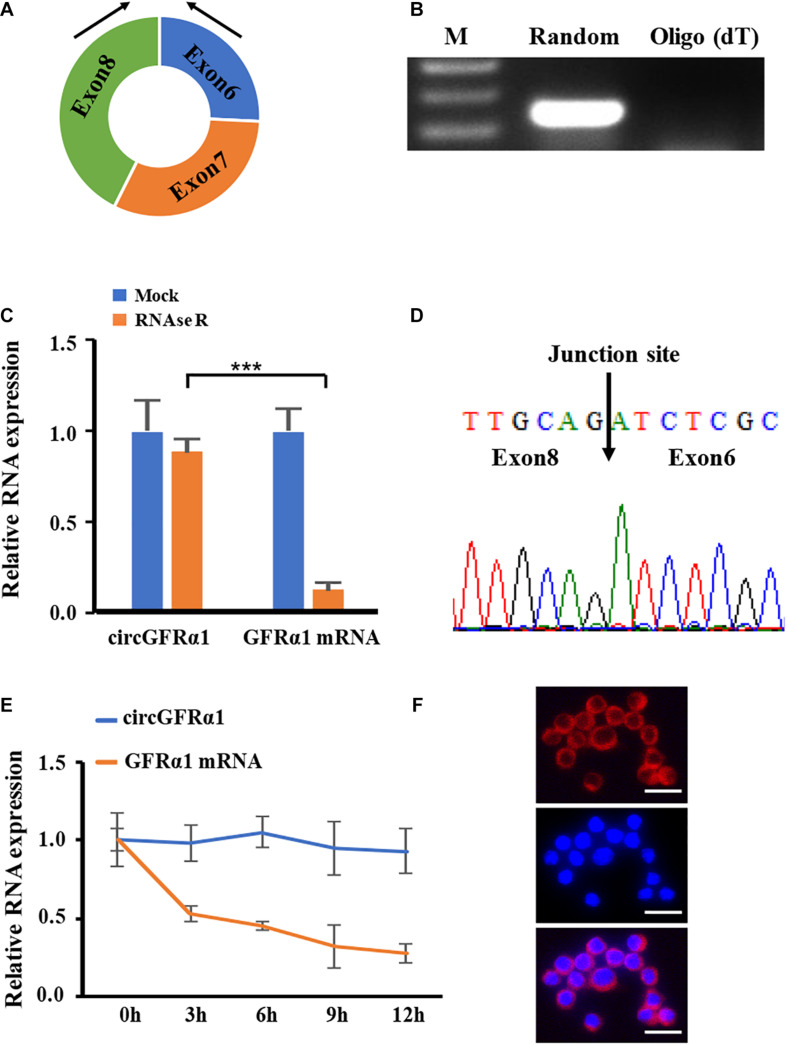
Characterization of circGFRα1 in FGSCs. **(A)** The genomic locus of circGFRα1. **(B)** RT-PCR products showing circularization of circGFRα1 with divergent primers. **(C)** After RNase R treatment in FGSCs, qRT–PCR showing the expression of circGFRα1 and GFRα1 mRNA. **(D)** Sanger sequencing of circGFRα1 demonstrating the head-to-tail splicing. **(E)** After Actinomycin D treatment, qRT–PCR showing the expression of circGFRα1 and GFRα1 mRNAs at the indicated time points. **(F)** RNA FISH for circGFRα1. Scale bars, 20 μm. ***P < 0.001.

To identify circRNAs involved in FGSC formation, we reanalyzed our previous circRNA expression data (Li et al., 2019) and found that circRNA_12447 (chr19: 58263912–58270163) is upregulated in FGSCs that possibly leads to FGSC differentiation and self-renewal. As discovered using the mouse reference genome (mm10), circRNA_12447 originated from exons 6–8 at the locus of GDNF family receptor alpha 1 (GFRα1) (Figure 1A) and was thus referred to as circGFRα1. For investigating the expression profile of circGFRα1 and verifying its circular shape, RT-PCR was performed using divergent primers, through which the expression of circGFRα1 in FGSCs was detected (Figure 1B). To confirm the circular shape of circGFRα1, RNase R, a 3′–5′ exoribonuclease with high process ability and no effect on circRNAs, was used. circGFRα1 developed resistance to RNase R exposure compared with the control linear gene GFRα1 (Figure 1C). In addition, RT-PCR products obtained after amplification with divergent primers were subjected to Sanger sequencing, which confirmed that circGFRα1 contains the back-spliced junction (Figure 1D). After exposure of circGFRα1 and the control linear gene to actinomycin D, a transcription inhibitor, our qRT-PCR results revealed that half-life of circGFRα1 is over 12 h, whereas that of the related linear transcript was only 3 h (Figure 1E), indicating higher stability of circGFRα1 in FGSCs. Furthermore, we performed fluorescence *in situ* hybridization assays that revealed that circGFRα1 is mostly located in the cytoplasm (Figure 1F). Taken together, the findings indicate the stable and rich expression of circGFRα1 in FGSCs.

### CircGFRα1 Affects Self-Renewal and Survival of Female Germline Stem Cells

**FIGURE 2:**
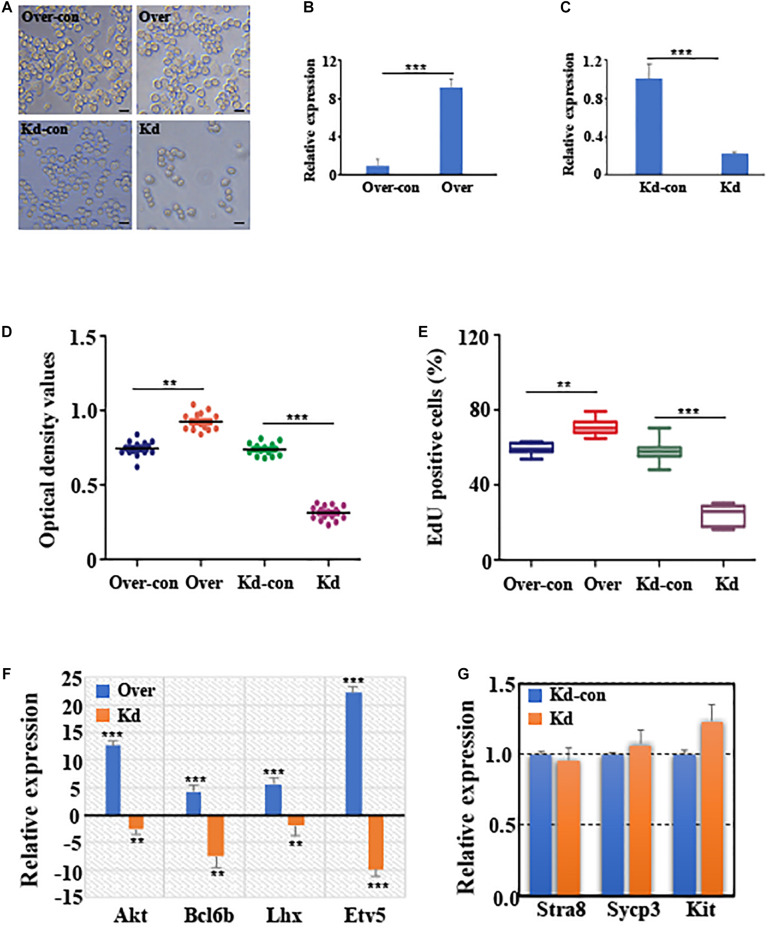
CircGFRα1 affects self-renewal and survival of FGSCs. **(A)** Selected images for FGSCs infected with lentivirus. **(B)** qRT-PCR analyses detected the RNA level of circGFRα1 in cells infected with the circGFRα1 overexpression lentivirus control (over-con), circGFRα1 overexpression lentivirus (over). **(C)** qRT-PCR analyses detected the RNA level of circGFRα1 in cells infected with the circGFRα1 knockdown lentivirus control (kd-con), circGFRα1 knockdown lentivirus (kd). **(D)** CCK-8 assays were conducted using FGSCs infected with the circGFRα1 overexpression lentivirus control (over-con), circGFRα1 overexpression lentivirus (over), circGFRα1 knockdown lentivirus control (kd-con), circGFRα1 knockdown lentivirus (kd). **(E)** EDU assays were conducted using FGSCs infected with the circGFRα1 overexpression lentivirus control (over-con), circGFRα1 overexpression lentivirus (over), circGFRα1 knockdown lentivirus control (kd-con), circGFRα1 knockdown lentivirus (kd). **(F)** Relative expression of Akt, Bcl6b, Lhx, and Etv5 in FGSCs after circGFRα1 overexpression (over) and knockdown (kd). Overexpression control and knockdown control as blank control groups respectively. When calculating the relative expression, the expression of control group was set as 1. A positive value indicates up regulation, and a negative value indicates down regulation. **(G)** Relative expression of Stra8, Sycp3, and Kit, FGSCs infected with the circGFRα1 knockdown lentivirus control (kd-con), circGFRα1 knockdown lentivirus (kd). **P < 0.01 and ***P < 0.001.

Tissue-specific expression results revealed that circGFRα1 is highly expressed in the mouse ovary (Supplementary Figure 1A). Moreover, the circGFRα1 level was found to be stage-specific during the development of mouse FGSCs; a high level of transcript was present in FGSCs, whereas significantly lower levels were detected in germinal vesicle (GV)-stage oocytes (*P* < 0.05) and metaphase II (MII)-stage oocytes (Supplementary Figure 1B, *P* < 0.001). To examine the effect of circGFRα1 on FGSC development, we regulated its expression through RNA interference or overexpression by inducing lentivirus infection (Figure 2A). As expected, circGFRα1 overexpression was found to significantly upregulate its level (Figure 2B, *P* < 0.001). However, relative to negative controls, its expression level was significantly decreased in shRNA-loaded lentivirus-infected FGSCs, which specifically bind to the circGFRα1 junction site (Figure 2C, *P* < 0.001). Then, we examined the effects of circGFRα1 overexpression and knockdown on FGSC proliferation through CCK-8 and EdU incorporation assays, respectively. According to CCK8 assay results, the OD values of FGSCs with circGFRα1 overexpression markedly increased relative to controls, whereas those of circGFRα1-knockdown FGSCs evidently decreased compared with those of controls (Figure 2D, *P* < 0.001). Moreover, the EdU assay results indicated that EdU-positive FGSCs with circGFRα1 overexpression that were transfected with lentivirus were markedly more in number than controls (Figure 2E, *P* < 0.001). However, transfection with circGFRα1-knockdown lentivirus markedly decreased the number of EdU-positive FGSCs compared with that of controls (Figure 2E, *P* < 0.001). Thus, we found that genes, including Akt, Bcl6b, Lhx, and Etv5, associated with self-renewal with the highest responsiveness to GDNF signaling within FGSCs are significantly upregulated (Figure 2F). Meanwhile, we found that the expression levels of genes associated with FGSC self-renewal were significantly downregulated (Figure 2F), whereas the expression levels of genes associated with differentiation, such as Stra8, Sypc3, and Kit, were extremely low and not affected by circGFRα1 knockdown (Figure 2G, *P* > 0.05). In summary, these findings suggest that the overexpression of circGFRα1 promoted the self-renewal and maintenance of FGSCs, while its knockdown impaired these characteristics.

### CircGFRα1 Serves as a Sponge for miR-449

**FIGURE 3:**
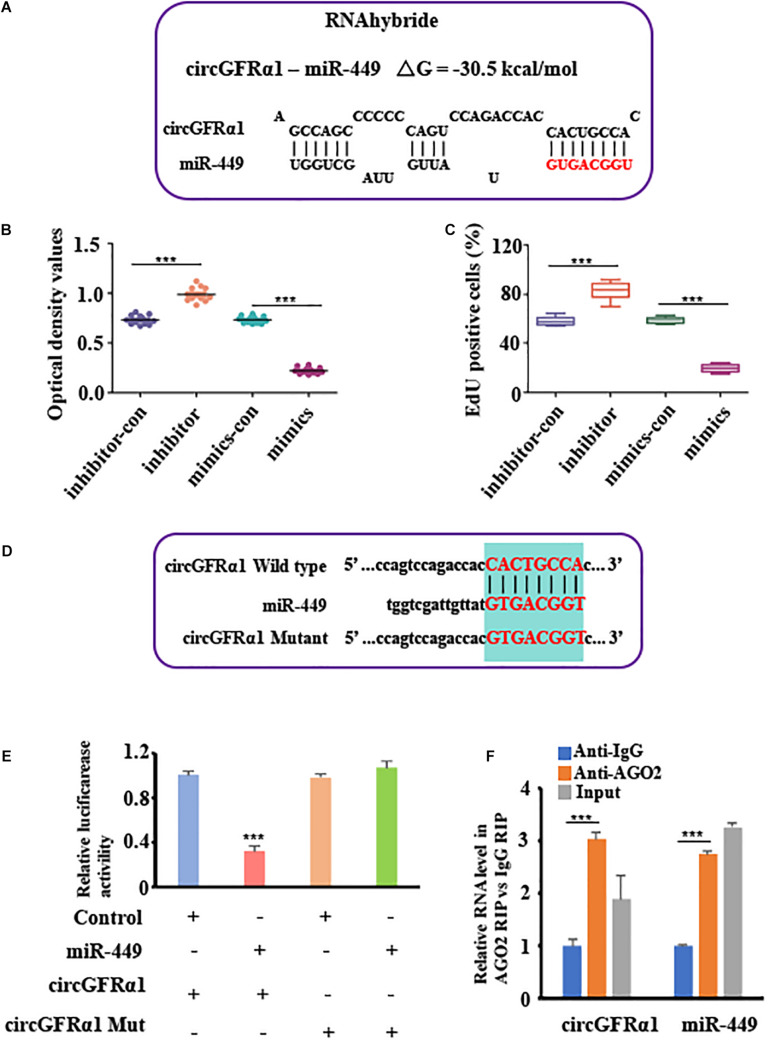
CircGFRα1 serves as a sponge for miR-449. **(A)** RNA hybrid analyses showed putative miR-449 binding sites in CircGFRα1. **(B)** CCK-8 assays were conducted using FGSCs infected with the miR-449 mimics control (mimics-con), miR-449 mimics (over), miR-449 inhibitor control (inhibitor-con), and miR-449 inhibitor (inhibitor). **(C)** EdU assays were conducted using FGSCs infected with the miR-449 mimics control (mimics-con), miR-449 mimics (over), miR-449 inhibitor control (inhibitor-con), and miR-449 inhibitor (inhibitor). **(D)** Target region of the CircGFRα1 for miR-449 and the mutant type of circGFRα1. **(E)** Effects of miR-449 on the activity of firefly luciferase reporters containing either circGFRα1 or mutant type circGFRα1 were assessed by luciferase reporter gene assays. **(F)** RIP assays were performed to detect miR-449 and circGFRα1. ***P < 0.001.

To explore the potential of circGFRA1 as a miRNA sponge, we performed the RNA hybrid analysis^1^. From our results, we predicted that certain miRNA-binding sites are present in circGFRα1. Of the estimated miRNAs, miR-449, which was also predicted to target the GFRα1 gene based on TargetScan and miRanda, was identified. Figure 3A shows the miR-449 seed region nucleotides (denoted in red). Later, CCK-8 and EdU assays were performed for examining the biological effects of miR-449 on FGSCs. We found that FGSCs transfected with miR-449 inhibitors have enhanced proliferation capacity, whereas those transfected with miR-449 mimics have markedly reduced proliferation capacity (Figures 3B,C). These findings indicate the role of miR-449 in the regulation of FGSC proliferation.

To verify whether the circGFRα1 transcript interacts with miR-449, the luciferase reported gene assay was performed using the circGFRα1-fused reporter gene (pGL3-circ GFRα1). In addition, a construct containing a non-specific circGFRα1 sequence (pGL3-circEGFR-MUT) and a wild-type construct (pGL3-circGFRα1-WT) were developed (Figure 3D). Then, miR-449 mimic and pGL3-circGFRα1-WT were co-transfected into the cells, which markedly reduced the luciferase activity in these cells compared with that in cells co-transfected with control miRNA and pGL3-circGFRα1-WT or co-transfected with mimic and pGL3-circGFRα1-MUT (Figure 3E). These findings indicated that miR-449 directly binds to circGFRα1 and adversely targets the latter. Furthermore, to verify whether circGFRα1 directly binds to miR-449, RIP assays were performed using control IgG or anti-AGO2 antibodies. The qRT-PCR assay was used to analyze miR-449 and circGFRα1. We found that anti-AGO2 antibodies markedly downregulate miR-449 and circGFRα1 compared with control IgG (Figure 3F), which suggests that miR-449 directly binds to circGFRα1 in the presence of AGO2.

### CircGFRα1 Acts as a Decoy of miR-449 to Upregulate Their Common Target, GFRα1

**FIGURE 4:**
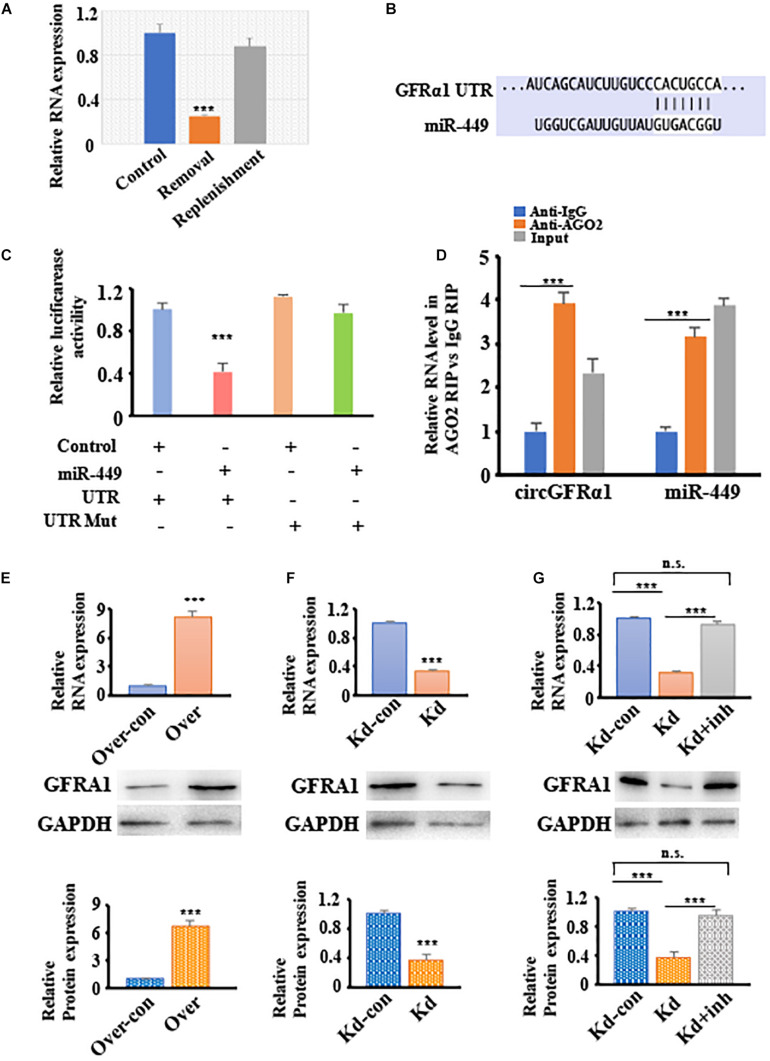
CircGFRα1 functions as a ceRNA to sequester miR-449 and upregulate the expression of GFRα1. **(A)** qRT–PCR analysis for the expression of circGFRα1 after GDNF removal and replenishment. **(B)** Target region of the 3′-UTR GFRα1 for miR-449. **(C)** Effects of miR-449 on the activity of firefly luciferase reporters containing either 3′-UTR GFRα1 or mutant type 3′-UTR GFRα1 were assessed by luciferase reporter gene assays. **(D)** RIP assays were performed to detect miR-449 and 3′-UTR GFRα1. **(E)** qRT–PCR and western blotting analysis for the expression of GFRα1 in FGSCs infected with the circGFRα1 overexpression lentivirus control (over-con), circGFRα1 overexpression lentivirus (over). **(F)** qRT–PCR and western blotting analysis for the expression of GFRα1 in FGSCs infected with the circGFRα1 knockdown lentivirus control (kd-con), circGFRα1 knockdown lentivirus (kd). **(G)** qRT–PCR and western blotting analysis for the expression of GFRα1 in FGSCs infected with the circGFRα1 knockdown lentivirus control (kd-con), circGFRα1 knockdown lentivirus (kd), and co-transfected miR-449 inhibitor and circGFRα1 knockdown lentivirus (kd + inhibitor). ***P < 0.001. n.s., no significant.

To explore the potential of circGFRα1 as a ceRNA for sequestering miR-449 and upregulating GFRα1 expression and activation of the GDNF signaling pathway. We firstly showed that the circGFRα1 expression is comparable to the GDNF signal after removal and replenishment of GDNF; its expression decreased 18 h after GDNF removal but increased after GDNF replenishment (Figure 4A). Moreover, TargetScan was used to identify the miR-449 putative target genes, which predicted GFRα1 (Figure 4B). To confirm this prediction, luciferase assays were performed using a GFRα1-fused reporter gene. Co-transfection of miR-449 mimic with the GFRα1 UTR significantly reduced the luciferase activity compared with that in control miRNA co-transfected with the GFRα1 UTR or in mimic co-transfected with the GFRα1 UTR (Figure 4C). These findings revealed that miR-449 directly binds to the GFRα1 UTR and adversely targets the latter. To verify whether the GFRα1 UTR directly binds to miR-449, RIP assays were performed using control IgG and anti-AGO2 antibodies; miR-449 and the GFRα1 UTR were analyzed through qRT-PCR. We found that anti-AGO2 antibodies markedly downregulate the expression of miR-449 and the GFRα1 UTR compared with control IgG (Figure 4D), indicating that the GFRα1 UTR directly binds to circGFRα1 depending on the presence of AGO2. To examine whether circGFRα1 regulates the GFRα1 level by interacting with miR-449, we detected GFRα1 expression in circGFRα1-deleted or circGFRα1-overexpressed FGSCs. As shown in Figures 4E,F, circGFRα1 overexpression increased the GFRα1 mRNA and protein expression, whereas circGFRα1 silencing reduced the GFRα1 mRNA and protein expression. Furthermore, the miR-449 inhibitor was co-transfected with si-circGFRα1 in FGSCs, which reversed the repression (Figure 4G). Taken together, the findings support the assumption that circGFRα1 modulates the GFRα1 level by directly interacting with miR-449.

### METTL14 Promotes Cytoplasmic Export of m^6^A-Modified circGFRα1 Through the GGACU Motif

**FIGURE 5:**
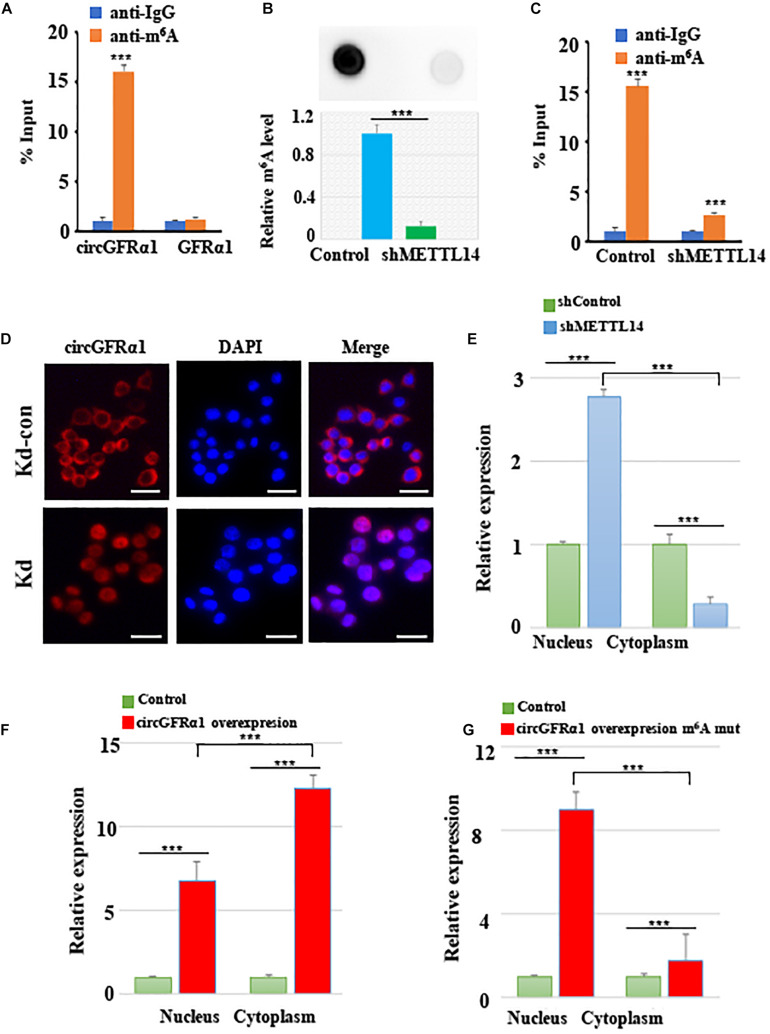
METTL14 promotes cytoplasmic export of m^6^A methylated circGFRα1. **(A)** MeRIP assay showing that m^6^A was highly enriched in circGFRα1 **(B)** Relative m^6^A level of FGSCs after METTL14 knockdown (kd). **(C)** MeRIP assay showing that down- regulation of METTL14 resulted in the decreased m^6^A level of circGFRα1. **(D)** RNA-FISH showing that the increased nuclear staining of circGFRα1 caused by METTL14 knockdown. **(E)** Cytoplasmic and Nuclear RNA Fractionation assay showing that knockdown of METTL14 increased the nuclear circGFRα1 content. **(F,G)** Cytoplasmic and Nuclear RNA Fractionation assay showing that the nuclear and cytoplasmic circGFRα1 contents were both increased, and mainly in nuclear fraction when mutated the GGACU m^6^A motif in circGFRα1 overexpressing construct. Scale bars, 20 μm. ***P < 0.001.

As circGFRα1 functioned as a ceRNA, it might be exported to the cytoplasm in an m^6^A-dependent manner. To study whether m^6^A modification occurs in circGFRα1, we first predicted the m^6^A sites using an online bioinformatic tool, m6Avar^2^, and found three RRACU m^6^A sequence motifs located in circGFRα1. Next, we performed methylated RNA immunoprecipitation (Me-RIP)–qPCR assays and found that the m^6^A level of circGFRα1 in FGSCs was very high (Figure 5A). We then performed shRNA-mediated silencing of METTL14, a core component of the m^6^A methylase complex, and found that downregulation of METTL14 resulted in decreases in the m^6^A levels of both total RNA and circGFRα1 (Figures 5B,C). The results of METTL14 knockdown were confirmed by western blotting (Supplementary Figure 2A). We then investigated whether m^6^A modification could affect the RNA metabolism of circGFRα1. Knockdown of METTL14 did not lead to a change in the expression of circGFRα1 (Supplementary Figure 2B).

In addition, we performed FISH and cytoplasmic and nuclear mRNA fractionation experiments, and found that silencing of METTL14 significantly increased the nuclear circGFRα1 content (Figures 5D,E, *p* < 0.001). We also found that, when circGFRα1 was overexpressed, both nuclear and cytoplasmic circGFRα1 levels were increased, particularly in the cytoplasmic fraction (Figure 5F, *p* < 0.001). By browsing the junction sequence in circGFRα1, we identified that the GGACU motif was a putative m^6^A motif. Once we mutated the GGACU m^6^A motif in a circGFRα1-overexpressing construct, although both nuclear and cytoplasmic circGFRα1 levels were increased, the main increase occurred in the nuclear fraction (Figure 5G, *p* < 0.001). These findings indicated that m^6^A modification of circGFRα1 facilitated circGFRα1 export from the nucleus to the cytoplasm in an m^6^A-dependent manner and that m^6^A modification of circGFRα1 are important for FGSC development.

## Discussion

**FIGURE 6:**
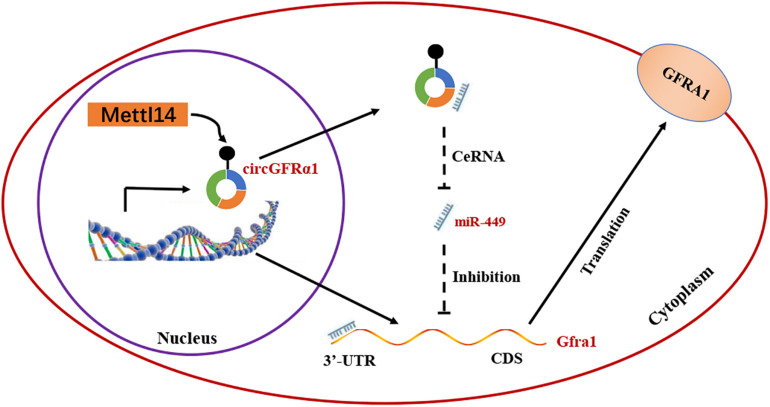
Proposed model of novel circGFRα1 exports to the cytoplasm mediated by METTL14, and promotes self-renewal of FGSCs. METTL14 alters the m^6^A modification of circGFRα1 and increases its export to the cytoplasm, while circGFRα1 promotes FGSC self-renewal by acting as a ceRNA that sponges miR-449, leading to enhanced GFRα1 expression and activation of the GDNF signaling pathway, and finally affecting self-renewal of FGSCs.

On the basis of the above findings, we propose a model (Figure 6) in which METTL14 alters the m^6^A modification of circGFRα1 and increases its export to the cytoplasm, while circGFRα1 promotes FGSC self-renewal by acting as a ceRNA that sponges miR-449, leading to enhanced GFRα1 expression and activation of the GDNF signaling pathway, finally affecting the development of FGSCs.

Recently, the role of circRNAs has become evident in determining the fate of FGSCs (Li et al., 2017a, 2019). CircRNAs, a newly discovered class of non-coding RNAs, have a covalent bond that links the 3′- and 5′-ends produced through back-splicing (Memczak et al., 2013). The expression of circRNAs is tissue- and stage-specific, and a small portion of circRNAs is highly conserved among different species (Salzman et al., 2013). Functional research on circRNAs has been focused mostly on nuclear transcriptional regulators, miRNA sponges, and RNA-binding proteins (Hansen et al., 2013). In this study, we discovered that miR-449 binds to GFRα1 and circGFRα1, indicating that circGFRα1 possibly plays the role of an miR-449 sponge in regulating the GFRα1 level through the ceRNA mechanism. Therefore, we suggest that circGFRα1 plays the role of a ceRNA for GFRα1 in FGSCs and serves as a miR-449 sponge. First, our bioinformatics analysis results demonstrated the presence of miR-449-binding sites in the 3′-UTR of both circGFRA1 and GFRA1. Second, these results were further validated through luciferase reporter assays. Third, circGFRα1 deletion downregulated the GFRα1 level. Finally, the inhibition of miR-449 reversed the above expression trend.

Increasing evidence has shown that m^6^A modification plays an important role in mammalian biology. For example, it is involved in upregulation of RNA stability (Wang et al., 2014), localization (Fustin et al., 2013), transport, cleavage (Molinie et al., 2016), and translation (Meyer et al., 2015) at the post-transcriptional level. Alarcon et al. (2015) found that Mettl3-dependent pri-miRNA methylation can promote DGCR recognition and processing, thus promoting microRNA maturation. In addition, HNRNPA2B1, an m^6^A recognition protein, promotes the processing of pri-miRNA into pre-miRNA (Alarcon et al., 2015). Moreover, the modification of circRNA can promote its translation (Yang et al., 2017). Yang et al. (2018) found that m^6^A-modified lincRNA 1,281 mediates the regulatory mechanism of ceRNA. Here, we found that m^6^A is enriched on circGFRα1 in FGSCs. Modification of m^6^A in circGFRα1 leads to the improvement of its RNA stability, which may partially account for the upregulation of circGFRα1 in FGSCs. In addition to m^6^A modification, other mechanisms might also be involved in the elevation of circGFRα1, such as DNA methylation, histone modification, and miRNA dysregulation, which warrant further exploration.

GDNF signal regulates the protein phosphorylation of downstream substrates by affecting the activity of protein kinases (PI3K–Akt, mitogen/ERK kinase, and Src family kinases, etc.), ultimately affecting the level of protein expression, which is the most important mechanism regulating SSC self-renewal and differentiation (Brinster and Avarbock, 1994; Oatley et al., 2007; He et al., 2008). GFRα1 is the receptor of GDNF, and GDNF can regulate the fate of stem cells only by binding with this receptor. Our previous study showed that GFRα1 was expressed on the surface of FGSCs, and that FGSCs had a mechanism of self-renewal involving GDNF similar to that of SSCs (Li et al., 2019). In this study, we found that circGFRα1 acts as a decoy of miR-449 to upregulate their common target, GFRα1.

Taking our findings together, we found that circGFRα1 promotes FGSC self-renewal by acting as a ceRNA that sponges miR-449, leading to enhanced GFRα1 expression and activation of the GDNF signaling pathway. Furthermore, circGFRα1 acts as a ceRNA based on the METTL14-mediated cytoplasmic export through the GGACU motif. Our study should help to understand the mechanisms regulating germ cell development, add new evidence on the mechanisms of action of circRNA, and clarify female reproductive mechanisms to improve the quantity and quality of oocytes.

## Data Availability Statement

The original contributions presented in the study are included in the article/Supplementary Material, further inquiries can be directed to the corresponding author/s.

## Author Contributions

XL conducted all the major experiments, data analysis, and wrote the manuscript. GT performed analysis with RNA Hybrid. JW initiated and supervised the entire project, analyzed data, and wrote the manuscript. All authors reviewed the manuscript and contributed in their areas of expertise.

## Conflict of Interest

The authors declare that the research was conducted in the absence of any commercial or financial relationships that could be construed as a potential conflict of interest.

## Footnotes

1 https://bibiserv.cebitec.uni-bielefeld.de/rnahybrid/

2 http://m6avar.renlab.org/
