# Supplementary material for: Novel circGFRα1 Promotes Self-Renewal of Female Germline Stem Cells Mediated by m6A Writer METTL14
Source: Front Cell Dev Biol. 2021 Apr 12;9:640402. doi: 10.3389/fcell.2021.640402 (PMC8076159; doi:10.3389/fcell.2021.640402)
Supplement: Supplementary file 1 [file Data_Sheet_1.PDF]

SUPPLEMENTARY MATERIAL FOR

Novel circGFRα1 promotes self-renewal of female germline stem cells mediated by m^{6}A writer METTL14

Xiaoyong Li[1], Geng Tian[1], Ji Wu[1,2,*]

[1] Renji Hospital, Key Laboratory for the Genetics of Developmental and Neuropsychiatric Disorders (Ministry of Education), Bio-X Institutes, School of Medicine, Shanghai Jiao Tong University, Shanghai, China

[2] Key Laboratory of Fertility Preservation and Maintenance of Ministry of Education, Ningxia Medical University, Yinchuan, China

[*]**Correspondence**:

Ji Wu

jiwu@sjtu.edu.cn

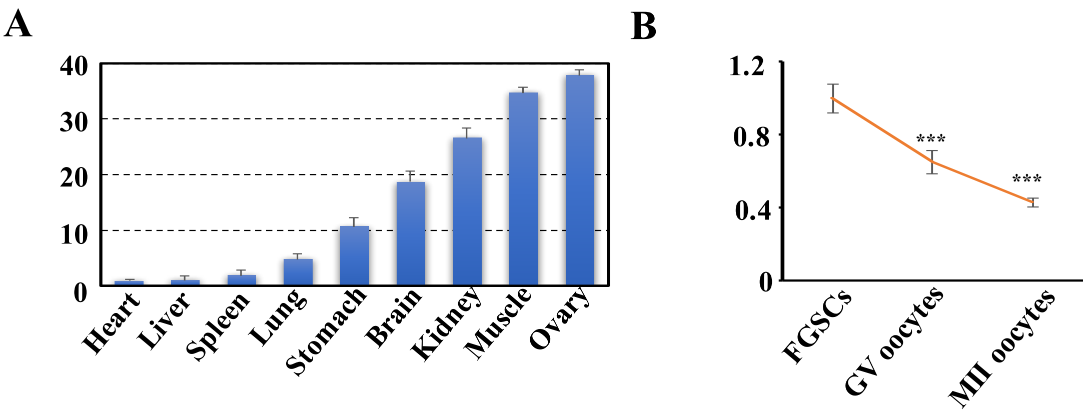

Supplementary Figure S1 | CircGFRα1 is highly abundant in mouse ovary and stage specific expression in mouse FGSCs development. **(A)** circGFRα1 RNA levels in different tissues were measured by qRT-PCR. **(B)** circGFRα1 RNA levels in FGSCs, PS, GV oocyres and MII oocytes were measured by qRT-PCR. C, qRT-PCR analyses detected the RNA level of circGFRα1 in cells infected with the circGFRα1 overexpression lentivirus control (over-con), circGFRα1 overexpression lentivirus (over). ***P < 0.001.

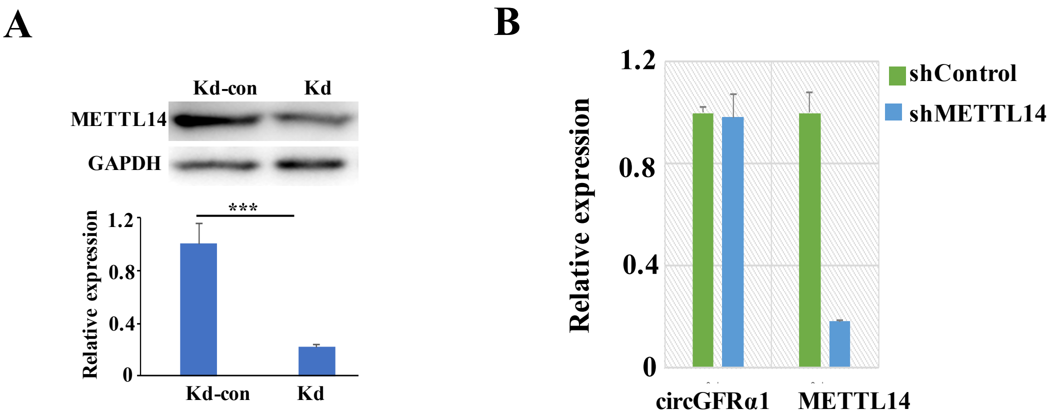

Supplementary Figure S2 | Knockdown of METTL14 does not lead to expression change of circGFRα1. **(A)** Western blot analyses detected the protein level of METTL14 in cells infected with the METTL14 knockdown lentivirus control (kd-con), METTL14 knockdown lentivirus (kd). **(B)** Knockdown of METTL14 does not lead to expression change of circGFRα1. ***$P < 0.001$.
